# The association of quality contraceptive counseling measures with postabortion contraceptive method acceptance and choice: results from client exit interviews across eight countries

**DOI:** 10.1186/s12913-022-08851-0

**Published:** 2022-12-13

**Authors:** Valerie N. Acre, Sally Dijkerman, Lisa M. Calhoun, Ilene S. Speizer, Cheri Poss, Ernest Nyamato

**Affiliations:** 1 Ipas, Chapel Hill, NC USA; 2 Carolina Population Center, University of North Carolina at Chapel Hill, Chapel Hill, NC USA; 3 Department of Maternal and Child Health and Carolina Population Center, University of North Carolina at Chapel Hill, Chapel Hill, NC USA

**Keywords:** Contraception, Contraceptive choice, Abortion, Quality of contraceptive services, Contraceptive counseling

## Abstract

The availability of a variety of modern contraceptive methods is necessary but insufficient to provide a high-quality contraceptive service to postabortion clients. Women, especially young women, must be empowered to make informed choices about which methods they receive, including whether to use contraception following an abortion service. In this study, we conducted 2,488 client exit interviews with abortion clients after their induced abortion service or postabortion care visit in Ipas-supported health facilities in eight countries: Argentina, Bolivia, Ethiopia, Kenya, Mexico, Nepal, Nigeria, and Uganda. We evaluated the quality of postabortion contraceptive counseling across two domains of contraceptive counseling: information exchange and interpersonal communication. We measured the association between these quality elements and two outcomes: 1) client-perceived choice of contraceptive method and 2) whether or not the client received a modern contraceptive method. We examined these relationships while adjusting for sociodemographic and confounding variables, such as the client feeling pressure from the provider to accept a particular method. Finally, we determined whether associations identified differ by age group: under 25 and 25+. Information exchange and interpersonal communication both emerged as important counseling domains for ensuring that clients felt they had the ability to choose a contraceptive method. The domain of information exchange was associated with having received a contraceptive method for all abortion clients, including young abortion clients under 25. Nearly 14% of clients interviewed reported pressure from the provider to accept a particular contraceptive method; and pressure from the provider was significantly associated with a client’s perception of not having a choice in selecting and receiving a contraceptive method during her visit to the facility. Improving interpersonal communication, strengthening contraceptive information exchange, and ensuring clients are not pressured by a provider to accept a contraceptive method, must all be prioritized in postabortion contraceptive counseling in health facilities to ensure postabortion contraceptive services are woman-centered and rights-based for abortion clients.

**Supplementary Information:**

The online version contains supplementary material available at 10.1186/s12913-022-08851-0.

## Background

Women and girls have the right to the highest attainable standard of health; this includes the right to four essential elements: availability, accessibility, acceptability, and quality (AAAQ). This AAAQ framework has been adopted and applied by various UN Treaty Monitoring Bodies to sexual and reproductive health, including abortion and contraceptive services [1]. Beginning with Bruce’s 1990 framework [2], the family planning and reproductive health community has paid particular attention to defining aspects of quality in contraceptive service delivery. Building off of Bruce’s framework, Jain and Hardee introduced a modified quality framework to respond to the differing articulations of quality in the context of human rights-based family planning [3].

In Jain and Hardee’s framework, appropriate information exchange and interpersonal relations are identified as important elements of contraceptive counseling. Information exchange is the communication of essential and appropriate information to clients to ensure they select an appropriate method that is both tailored to their needs and circumstances which enables effective contraceptive use. Interpersonal communication and relations (henceforth referred to as interpersonal communications) refers to the formation of a positive therapeutic relationship between the provider and client in which clients are treated with dignity and respect [3].

In practice, it has been shown that interpersonal communication [4–6] and information exchange [7–9] are often lacking during contraceptive counseling. Instead, provider-dominated approaches to contraceptive counseling are common, allowing little engagement between clients and providers when clients are selecting a method [4–6]. This can result in a lack of tailored counseling that is responsive to clients’ needs and preferences [6, 10]. Additionally, studies in both high-income and low-income settings have found varying levels of coercion in contraceptive counseling [11–15]. For example, in research undertaken in an anonymous sub-Saharan country, Senderowicz identified experiences of overt and subtle forms of coercion towards clients by service providers to accept any contraceptive method or to accept a particular method during provision of contraceptive services [13]. The ability to choose a contraceptive method is not only paramount for rights-based service provision, it also has been shown, across a variety of settings in low- and middle-income countries (LMIC), to improve satisfaction with care which, in turn, supports the clients’ contraception adoption and continuation [4, 16–18].

For abortion clients, contraceptive counseling is an important component of quality care yet is commonly overlooked [19, 20]. Contraceptive counseling is particularly important for abortion clients because abortion clients have specific counseling needs and experience additional barriers to care. For example, abortion clients should be informed that their fertility can return quickly after her abortion, before their next menstrual period [21]. Evidence shows that contraceptive counseling provided to abortion clients often falls short, lacking personalization to their specific life circumstances [22].

Abortion clients also face additional scrutiny resulting from the stigma surrounding sexuality and abortion [19, 20], especially young and unmarried people [23, 24]. Abortion stigma may also result in providers pressuring abortion clients to accept contraception, or a particular contraceptive method, yet evidence from LMICs is lacking. Filling this evidence gap by assessing the quality of counseling for abortion clients is critical to address these issues.

In this study, we examine the quality of contraceptive counseling provided to abortion clients at Ipas-supported health facilities across eight countries: Argentina, Bolivia, Ethiopia, Kenya, Mexico, Nepal, Nigeria, and Uganda. The countries included in the study have varying laws and policies concerning abortion access (see Table 1) [25]. Ipas is an international organization focused on fulfillment of sexual and reproductive rights by expanding access to abortion and contraceptive care. Globally, Ipas programs provide clinical training, mentorship, and programmatic support to healthcare workers providing comprehensive abortion care (CAC) in health facilities, including postabortion contraceptive care. CAC services encompass people seeking either induced abortion or postabortion care (PAC). Induced abortion clients present at the health facility pregnant and receive a series of interventions from health care professionals to induce their abortion. PAC clients present at the health facility with an incomplete abortion that began before their arrival at the health facility and receive a series of interventions to manage and complete the abortion. Underlying Ipas’s model is a client-centered approach to CAC to allow each woman to exercise her right to reproductive autonomy, as outlined in the Ipas Woman-Centered, Comprehensive Abortion Care (CAC) manual [26].

**Table 1:** Abortion laws and policies in Ipas intervention countries

Country	Country’s Abortion Law as of January 2022
Argentina	Abortion is available upon request until 14 weeks gestation and is available for pregnancies that were a result of rape or incest, or to save the life or health of the pregnant person
Bolivia	Abortion is permitted in the cases of rape, incest, and mental health
Ethiopia	Abortion is permitted in cases of rape, incest, fetal impairment, to save the life or health of the pregnant person and if the pregnant person is unfit physical or mentally to care for a child
Kenya	Abortion is available to preserve the life or health of the pregnant person
Mexico	Abortion is available upon request until 12 weeks in Mexico City; laws in the states vary with a majority of states permitting abortion in cases of rape, fetal impairment and to save the life of the pregnant person
Nepal	Abortion is available upon request until 12 weeks and available for pregnancies until 28 weeks that were a result of rape, incest, or to save the life or health of the pregnant person
Nigeria	Abortion is available to save the life of the pregnant person
Uganda	Abortion is available to save the life of the pregnant person

The objectives of this study are to examine the association between two quality elements (interpersonal communication and information exchange) and 1) client-perceived choice to accept or not accept a contraceptive method and 2) whether or not the client received a contraceptive method. We further determine whether associations identified differ by two age groups: under 25 and 25+, using the World Health Organization definition for young people [27]. We hypothesize that clients receiving higher quality contraceptive counseling will be more likely to receive a contraceptive method and feel empowered to make their own choice to accept or not accept a contraceptive method. We also hypothesize that younger abortion clients receive lower quality contraceptive counseling and experience more pressure to accept a method than older clients.

## Methods

### Data

The data for this paper come from client exit interviews with women ages 15 to 49 who received induced abortion or postabortion care (PAC) between 2019 and 2020 at Ipas-supported healthcare facilities in Argentina, Bolivia, Ethiopia, Kenya, Mexico, Nepal, Nigeria, and Uganda. We employed a census or stratified random sample of facilities, our primary sampling unit, based on the program size in the country. The facility sampling strategy by country is shown in Table 2. Program size was defined as the number of CAC facilities receiving Ipas’s support in each country – very small: < 30 facilities, small: 30 to 100 facilities, medium: 100 to 300 facilities, and large: over 300 facilities. Stratified random sampling was used to select strata by type (primary, secondary, or tertiary) and Ipas-intervention region, totaling 3–5 facilities per stratum. Target sample sizes for clients ranged from 100 CAC clients in very small programs (< 30 facilities) to 900 clients in large programs (> 300 facilities). We sampled a minimum of one client at each participating health facility and collected data for a minimum of five consecutive days in order to meet the target sample sizes for clients and to capture potential variability in clients’ experiences (e.g., differences in clients’ experience resulting from rotation of providers within a facility). All abortion clients receiving care during the data collection period were recruited to participate in the interview after they had completed their service or care but before leaving the facility, assuming they consented and met eligibility criteria (country-specific age requirements and were in stable post-abortion health).

**Table 2:** Facility Sampling Strategy by Ipas intervention country

Country	Program Size	Facility Sampling Strategy	Number of facilities included
Argentina	Very small	Census	7
Bolivia	Very small	Census	31
Ethiopia	Large	Stratified random sample	114
Kenya	Small	Stratified random sample	53
Mexico	Very small	Census	25
Nepal	Medium	Stratified random sample	60
Nigeria	Medium	Stratified random sample	62
Uganda	Small	Census	23

All women interviewed were asked to provide verbal or written consent to participate depending on country context. The minimum client age for inclusion in client exit interviews varied by country. Minimum age inclusion was determined by the local Ipas office, participating ministries of health in each country, and/or local IRBs. The minimum age in Nepal, Kenya and Ethiopia was 14; in Mexico, Bolivia, Nigeria and Uganda it was 15, and in Argentina it was 18. Parental consent was required for women under the age of 18 in Mexico, Uganda, Kenya, and Nigeria, and required for women under the age of 17 in Nepal. Parental consent was waived for women under the age of 18 in Ethiopia and Bolivia. In Bolivia, parental consent was waived because adolescents of any age may request and receive sexual and reproductive health services (SRH) without the consent of their parents or guardian [28]. Given that in Bolivia adolescents are legally able to decide to receive SRH services, they are also able to decide to participate in an SRH-related study. In Ethiopia, parental consent was waived for unaccompanied women under the age of 18 because adolescent clients as young as 14 were considered emancipated minors. Ipas-trained interviewers conducted the interviews in spaces in a facility that could provide as much visual and auditory privacy as possible. Interviewers collected data by one of two ways in each country: 1) interviewers entered responses into a digital version of the questionnaire in Open Data Kit (ODK) on mobile devices (Argentina, Bolivia, Mexico, and Nigeria) or 2) interviewers wrote down responses on a paper questionnaire which was subsequently digitized (Ethiopia, Kenya, Uganda, and Nepal).

The questionnaire included four core sections across all countries – client background, the abortion service, the contraceptive service, and client feedback. The client background section included questions about the client’s age, education, relationship status and past use (or non-use) of contraception. The abortion service section included questions about the abortion service that the client received at the health facility. This section included questions like how many weeks pregnant the client was at the time of her service, whether she had an induced abortion or PAC service, the type of procedure she received, the types of information she received from the provider during her pre- and post-procedure counseling, and whether she had sufficient privacy during her abortion service. The contraceptive service section included questions about the contraceptive services the client received after the abortion service at the facility (if any). This section included questions about whether the client received contraceptive counseling, was asked about her fertility intentions, received pressure to choose a particular contraceptive method, and felt she had sufficient privacy during her contraceptive service. Lastly, the client feedback section included questions about her overall experience in the facility as well as questions about whether she was treated with dignity and respect during her visit, if she felt she could trust the staff to provide confidential care, whether she felt listened to, whether she would return to this facility for another service, and whether she would recommend this facility to a friend. Ipas used the information gathered from these interviews to inform health facility interventions and Ipas programming to strengthen abortion and contraceptive services.

### Dependent variables

The two main outcomes included 1) client-reported ability to choose the contraceptive method (or no method) of their choice and 2) client-reported receipt of a modern contraceptive method (condom, pills, emergency contraceptive pills, injectables, intrauterine contraceptive device (IUCD), implant or female sterilization), both measured as Yes = 1 and No = 0. Clients were asked “Did you feel like you were able to make the choice about your contraceptive method (or no contraceptive method) on your own?” with answer options of ‘yes’ and ‘no.’ Clients were asked “What contraceptive method did you receive today?” Response options included the full list of contraceptive methods (or no method).

### Independent variables

We created composite metrics to capture two aspects of quality of contraceptive services –information exchange and interpersonal communication. These two metrics were modeled after the Bruce [2] and Jain and Hardee [3] frameworks of quality of care for family planning services, and Dehlendorf and colleagues’ [29] categories of communication in the health care setting. The Ipas CAC training manual also highlights postabortion contraceptive counseling practices that align with these two quality-of-care domains [26].

Information exchange refers to the two-way communication between providers and clients to enable clients to choose and use contraception. The information exchange metric includes five yes/no questions on whether the client: 1) was asked about her desire to delay or prevent pregnancy, 2) understood the information given to her about contraceptive methods, 3) felt she was given enough information to choose a contraceptive method, 4) received information about a barrier method for protecting against sexually transmitted infections (STIs), and 5) received information about the quick return of fecundity unless using contraception. Interpersonal communication refers to the way clients are treated by providers, including treating clients with dignity and respect and maintaining privacy and confidentiality. The interpersonal communication metric includes five yes/no questions on whether the client: 1) was given the opportunity to ask questions about contraceptive methods, 2) felt she had enough privacy while talking to the provider about contraceptive options, 3) was treated with dignity and respect, 4) trusted the staff and providers to give her private, confidential care, and 5) felt that the provider listened to her needs and concerns. For each metric, an affirmative response to each item received 1 point and the points for each item were summed together with a possible score ranging between 0 and 5, with a score of 5 representing highest quality. Clients that failed to respond to one or more of the five questions from each metric were not given a score.

Clients were also asked whether or not the provider exerted pressure to select a particular method, and this item was another element of quality of care included in the analysis.

### Descriptive variables

Client sociodemographic characteristics, previous use of contraception, client fertility intentions, and abortion service characteristics were included in the survey. Client sociodemographic variables include the country in which the client received her abortion care, client age (< 25 years, 25 + years), relationship status (currently or formerly married, never married), and highest education level completed (none, primary, secondary, tertiary). Also included in the survey were her previous use of any contraception (modern or traditional) to delay or avoid getting pregnant (yes, no), and her fertility intentions: when she would like to have a or another child (within a year, between one and two years, more than two years, doesn’t want any more children, other). Client-reported characteristics of the abortion service received include gestational age of the pregnancy (less than 13 weeks gestation, at or after 13 weeks gestation), type of abortion service received (induced abortion, postabortion care), and abortion procedure method received (manual vacuum aspirator (MVA), medical abortion (MA), other surgical procedures).

### Analytic approach

Using Stata/SE 16.1, we undertook a descriptive analysis of sociodemographic characteristics, previous use of contraception, client fertility intentions, and abortion service characteristics of the full sample across the eight countries. We did a bivariate analysis of 1) receipt of contraceptive counseling by the characteristics previously mentioned, 2) the age of clients (split into two age categories of clients under 25 years old, and 25 +) by each outcome, and 3) the quality of contraceptive counseling elements by the two-category age of client. We used Chi-square, Fisher’s exact, and Wilcoxon-Mann-Whitney tests to assess the association between each independent and dependent variable, using a significance level of < 5%. We developed unadjusted and adjusted multivariable logistic regression models for the main outcomes (client-reported ability to choose the contraceptive method - or no method- of their choice and client-reported receipt of a modern contraceptive method) to assess the association with three quality-of-care elements: the contraceptive information exchange metric, the interpersonal communication metric, and provider pressure to accept a method. We report results as odds ratios (OR) and 95% confidence intervals (CI).

We adjusted for potential confounders (client background characteristics and client-reported characteristics of the abortion service received) in the analysis. The client background characteristics included are country, marital status, age group, education and having ever used contraception. The client-reported characteristics of the abortion service received include the gestational age of the pregnancy, the type of abortion service received, and the type of abortion procedure received.

Because facility identifiers were only available for six of the eight countries, we conducted a sensitivity analysis for each outcome to assess the influence of facility clustering in our six-country sample. This sensitivity analysis adjusts for the fact that the quality of care within a facility may be similar across clients from the same facility which may attenuate the results. After excluding clients from Nepal and Ethiopia, where unique facility identifiers were not available, the conclusions found with and without site-level clustering were similar across the six countries included. Given this, the results displayed in this paper include all countries without adjusting for site-level clustering. The results of the sensitivity analysis and cluster-adjusted analyses are available in Supplemental Table 1.

## Results

Table 3 presents the client demographic and abortion service characteristics for the total sample of abortion clients, as well as by whether the client received postabortion contraceptive counseling or not. A total of 2,488 abortion clients were interviewed; 56.3% (1,401 of 2,488) were PAC clients while 42.8% (1,065 of 2,488) were induced abortion clients with a majority (81.4%; 2,026 of 2,488) of services provided to clients with gestations under 13 weeks. Nearly half (44.5%; 1,108 of 2,488) of the sample were young clients under 25.

**Table 3:** Client Sociodemographics and Abortion Service Characteristics Overall, by Client Age Category, and by Receipt of Contraceptive Counseling

	All Clients (*N* = 2,488)		Bivariate Results of client sociodemographic and service characteristics by client age and receipt of contraceptive counseling			
			**Clients Age**		**Receipt of Postabortion Contraceptive Counseling**	
			**Under 25** **(** ***N*** **= 1,108)**	**25 and over** **(** ***N*** **= 1,380)**	**Yes** **(** ***N*** **= 2,097)**	**No** **(** ***N*** **= 388)**
	**n**	**%**	**%**	**%**	**%**	**%**
**Location**						
* Country* ^α, β^						
Argentina	115	4.6%	35.7%	64.3%	84.3%	15.7%
Bolivia	216	8.7%	43.1%	56.9%	81.0%	19.0%
Ethiopia	560	22.5%	54.3%	45.7%	94.3%	5.7%
Kenya	383	15.4%	60.3%	39.7%	83.8%	16.2%
Mexico	141	5.7%	43.3%	56.7%	80.9%	19.1%
Nepal	297	11.9%	28.6%	71.4%	91.2%	8.8%
Nigeria	650	26.1%	36.6%	63.4%	74.9%	25.1%
Uganda	126	5.1%	43.7%	56.3%	82.5%	17.5%
**Client Sociodemographics**						
* Age Category*						
Under 25	1108	44.5%			84.7%	15.3%
25 and over	1380	55.5%			83.9%	16.1%
* Relationship Status* ^α, β^						
Currently or formerly married	1600	64.3%	36.1%	63.9%	83.2%	16.8%
Never married	882	35.5%	60.1%	39.9%	86.3%	13.7%
Missing	6	0.2%	16.7%	83.3%	83.3%	16.7%
* Highest Education Level Completed* ^α, β^						
None	486	19.5%	25.5%	74.5%	79.0%	21.0%
Primary	488	19.6%	41.4%	58.6%	82.8%	17.2%
Secondary	1001	40.2%	56.2%	43.8%	87.1%	12.9%
Tertiary or higher	487	19.6%	41.9%	58.1%	85.2%	14.8%
Missing	26	1.0%	57.7%	42.3%	84.6%	15.4%
**Past Use of Contraception**						
* Ever used anything or tried in any way to delay or avoid getting pregnant previously* ^α, β^						
Yes	1433	57.6%	36.5%	63.5%	87.6%	12.4%
No	1025	41.2%	55.1%	44.9%	79.3%	20.7%
Missing	30	1.2%	66.7%	33.3%	96.7%	3.3%
**Fertility Intentions**						
* When she would like to have a/another child* ^α, β^						
Within a year	423	17.0%	43.5%	56.5%	76.4%	23.6%
Between one and two years	318	12.8%	41.8%	58.2%	87.1%	12.9%
More than two years	766	30.8%	59.9%	40.1%	90.5%	9.5%
Doesn’t want any more children	538	21.6%	14.9%	85.1%	84.6%	15.4%
Other	251	10.1%	51.8%	48.2%	80.5%	19.5%
Missing	192	7.7%	63.5%	36.5%	76.6%	23.4%
**Comprehensive Abortion Care (CAC) Service Characteristics**						
* Gestation by LMP* ^β^						
Less than 13 weeks gestation	2026	81.4%	44.8%	55.2%	85.6%	14.4%
At or after 13 weeks gestation	343	13.8%	41.1%	58.9%	74.6%	25.4%
Missing	119	4.8%	50.4%	49.6%	89.1%	10.9%
* Service type* ^α, β^						
Induced	1065	42.8%	52.4%	47.6%	91.0%	9.0%
PAC	1401	56.3%	38.5%	61.5%	79.1%	20.9%
Missing	22	0.9%	50.0%	50.0%	90.9%	9.1%
* Procedure Method* ^α, β^						
MVA	1332	53.5%	40.1%	59.9%	81.9%	18.1%
MA	996	40.0%	49.3%	50.7%	88.0%	12.0%
Other surgical procedure	131	5.3%	48.9%	51.1%	80.2%	19.8%
Missing	29	1.2%	65.5%	34.5%	86.2%	13.8%
**Receipt of Postabortion Contraceptive Counseling**						
* Received information about contraceptive methods*						
Yes	2097	84.3%	44.8%	55.2%		
No	388	15.6%	43.3%	56.7%		
Missing	3	0.1%	33.3%	66.7%		

All *P*-values reported from Chi-square test or Fisher’s Exact test for expected cell counts < 5

^α^ Represents a *p* < 0.05 for the bivariate result of the corresponding sociodemographic and service characteristics and client age category

^β^ Represents a *p* < 0.05 for the bivariate result of the corresponding sociodemographic service characteristic and receipt of postabortion contraceptive counseling

As shown in the middle column of Table 3, the percentage of young abortion clients under age 25 varied by a number of demographic and service characteristics. A greater proportion of never married abortion clients were young (60.1%; 530 of 882, *p* < 0.001). Additionally, clients who had never used anything to avoid getting pregnant previously were more likely to be young (55.1%, 565 of 1,025, *p* < 0.001), and clients who wanted to wait more than two years to have children were also more likely to be young (59.9%, 459 of 766, *p* < 0.001). With respect to abortion service characteristics, a greater percentage of induced abortion clients were young clients (52.4%; 558 of 1,065) compared to PAC clients (38.5%; 539 of 1,401).

Most abortion clients (84.3%; 2,097 of 2,488) received postabortion contraceptive counseling during their visit. As shown in the right-hand side of Table 3, the percentage of clients that received postabortion counseling varied by a number of demographic factors. For example, while 94.3% (528 of 560) of clients in Ethiopia and 91.2% (271 of 297) in Nepal received postabortion contraceptive counseling, only 74.9% (487 of 650) of clients in Nigeria reported the same (*p* < 0.001). There was no significant difference in the age of clients who received contraceptive counseling and those who did not. Service characteristics differed significantly between clients who received contraceptive counseling and those who did not. Clients with gestations at or after 13 weeks were less likely to receive contraceptive counseling compared to clients with gestations under 13 weeks (74.6%, 256 of 343, and 85.6%, 1,735 of 2,026, respectively, *p* < 0.001). PAC clients were less likely to receive contraceptive counseling compared to induced abortion clients (79.1%, 1,108 of 1,401, and 91.0%, 969 of 1,065, respectively, *p* < 0.001). Lastly, a quarter (24%; 92 of 388) of clients that did not receive contraceptive counseling reported receiving a contraceptive method after their abortion service. A majority of clients that received a method without counseling (55%; 51 of 92) received a short-term method, while 45% (41 of 92) received a long-term method: IUCD, implant, or sterilization (data not shown in tables). We discuss this finding further in the Discussion section.

Table 4 provides the descriptive findings of key contraceptive counseling variables used in the information exchange and interpersonal communication metrics, the scores of each metric, and whether the client felt pressure from the provider to accept a particular method, among clients who received contraceptive counseling. For this table and subsequent tables, only clients who received any contraceptive counseling were included in these analyses. Nearly half (47.2%, 950 of 2,097) of all clients received the maximum score of 5 in the information exchange metric. For four of the five items in this metric, over 90% of clients responded positively to each. However, only half of clients received information about barrier methods for protecting against STIs (53.4%, 1,094 of 2,097). Clients under 25 were more likely to receive information about barrier methods compared to clients 25+ (57.9%, 528 of 939, versus 49.9%, 566 of 1,158, *p* < 0.001). This difference results in the higher average information exchange score of 4.3 for young clients compared to their older counterparts, who have a score of 4.2 (*p* < 0.05).

**Table 4:** Descriptive findings on key provider and client interaction variables by age among clients counseled on contraception

			Age Groups			
	**All Clients Receiving Contraceptive Counseling (*N*** **= 2,097)**		**Under 25** **(** ***n*** **= 939)**		**25 and over** **(** ***n*** **= 1,158)**	
	**n**	**%**	**n**	**%**	**n**	**%**
*Contraceptive Information Exchange Metric Elements*						
Provider asked client about her desire to delay or prevent pregnancy (% yes)	1912	92.1%	848	91.4%	1064	92.6%
Missing	20		11		9	
Client understood the information given to her about contraceptive methods (% yes)	1957	94.1%	884	94.6%	1073	93.6%
Missing	17		5		12	
Client felt she was given enough information to choose a contraceptive method (% yes)	1882	90.7%	850	91.6%	1032	90.1%
Missing	23		11		12	
Client was told about a barrier method for protecting against STIs (% yes) ***	1094	53.4%	528	57.9%	566	49.9%
Missing	50		27		23	
Provider told client that without using a contraception method she could get pregnant again quickly, even before her next menstruation (% yes)	1917	93.4%	852	93.2%	1065	93.5%
Missing	44		25		19	
Contraceptive Information Exchange Metric Score *						
Mean Score (SD)	4.2 (0.9)		4.3 (0.9)		4.2 (0.9)	
0 of 5	4	0.2%	1	0.1%	3	0.3%
1 of 5	26	1.3%	11	1.2%	15	1.3%
2 of 5	87	4.3%	33	3.7%	54	4.8%
3 of 5	202	10.0%	101	11.2%	101	9.1%
4 of 5	745	37.0%	294	32.7%	451	40.5%
5 of 5	950	47.2%	460	51.1%	490	44.0%
Missing one or more component	83		39		44	
*Interpersonal Communication Metric Elements*						
Client was given the opportunity to ask questions about contraceptive methods (% yes)	1799	86.2%	799	85.2%	1000	87.0%
Missing	10		1		9	
Client felt she had enough privacy while talking to the provider about contraceptive options (% yes)	1882	92.2%	845	93.2%	1037	91.4%
Missing	55		32		23	
Client felt the staff and providers treated her with dignity and respect (% yes)	2030	97.3%	902	96.7%	1128	97.8%
Missing	11		6		5	
Client felt that she could trust the staff and providers to give her private, confidential care (% yes)	1956	93.9%	874	93.9%	1082	93.8%
Missing	13		8		5	
Client felt that the provider listened to her needs and concerns (% yes) *	1979	95.0%	874	93.7%	1105	96.1%
Missing	14		6		8	
*Interpersonal Communication Metric Score*						
Mean Score (SD)	4.6 (0.8)		4.6 (0.8)		4.7 (0.8)	
0 of 5	5	0.2%	4	0.4%	1	0.1%
1 of 5	19	0.9%	8	0.9%	11	1.0%
2 of 5	49	2.4%	25	2.8%	24	2.1%
3 of 5	97	4.8%	38	4.2%	59	5.3%
4 of 5	271	13.4%	131	14.6%	140	12.5%
5 of 5	1576	78.1%	693	77.1%	883	79.0%
Missing one or more component	80		40		40	
*Client felt pressure from the provider to accept a particular contraceptive method*						
Yes	284	13.7%	132	14.2%	152	13.2%
No	1793	86.3%	797	85.8%	996	86.8%
Missing	20		10		10	

Percentages shown exclude missing data

*P*-values reported from Chi-square test or Fisher’s Exact test for expected cell counts < 5 and Wilcoxon-Mann-Whitney test for continuous variables. *P*-values from bivariate analyses demonstrate differences between the provider and client interaction variables and client age group

* *p* < 0.05; ** *p* < 0.01; *** *p* < 0.001

Overall, 78.1% (1,576 of 2,097) of abortion clients received the maximum score of 5 on the interpersonal communication metric, and there were no differences in the score between the two age groups.

Table 5 provides the descriptive findings of the postabortion contraception outcomes both overall and by two age groups, among abortion clients who received contraceptive counseling. 89% (88.5%, 1,842 of 2,097) of clients felt able to make a choice about their contraceptive method with no difference by age group. Clients under 25 were more likely to have received a modern contraceptive method as compared to women age 25+, 75.3% (707 of 939) vs. 67.4% (780 of 1,158) (*p* < 0.001).

**Table 5:** Postabortion Contraceptive Outcomes by Age Group, among clients who received contraceptive counseling

	All Clients Receiving Contraceptive Counseling (*N* = 2,097)		Age Groups			
			**Under 25** **(** ***n*** **= 939)**		**25 and over** **(** ***n*** **= 1,158)**	
	**n**	**%**	**n**	**%**	**n**	**%**
*Client felt like she was able to make the choice about her contraceptive method (or no method)*						
Yes	1842	88.5%	817	87.8%	1025	89.0%
No	240	11.5%	113	12.2%	127	11.0%
Missing	15		9		6	
Client received a modern contraceptive method postabortion***						
Yes	1487	70.9%	707	75.3%	780	67.4%
No	610	29.1%	232	24.7%	378	32.6%
Missing	0					
*Contraceptive Method Received* ^a^ ***						
Condoms	113	5.4%	47	5.0%	66	5.7%
Oral pills	179	8.5%	71	7.6%	108	9.3%
Emergency Pills	4	0.2%	3	0.3%	1	0.1%
Contraceptive Patch	2	0.1%	1	0.1%	1	0.1%
Injectables	532	25.4%	251	26.7%	281	24.3%
Implant	503	24.0%	269	28.6%	234	20.2%
IUCD	143	6.8%	64	6.8%	79	6.8%
Female Sterilization	13	0.6%	2	0.2%	11	0.9%
No modern method	608	29.0%	231	24.6%	377	32.6%
Missing	0		0		0	0%

Percentages shown exclude missing data

^a^ For clients that received more than one modern contraceptive method (*n* = 4), the most effective method was selected.

*P*-values reported from Chi-square test or Fisher’s Exact test for expected cell counts < 5. *P*-values from bivariate analyses demonstrate differences between each outcome variable and client age group.

* *p* < 0.05; ** *p* < 0.01, *** *p* < 0.001

Table 6 provides the unadjusted and adjusted logistic regression odds ratios (OR) and 95% CI for the models of the client’s perception of making her own choice of contraceptive method (or choice of no method). This table only includes the main independent variables, but results for the sociodemographic variables were in expected directions (see Supplemental Table 2). For each point increase in the information exchange metric (i.e., with each additional element of counseling included by the provider), the adjusted odds of the abortion client reporting she was able to make a choice of contraceptive method increases by 140% (aOR = 2.4, *p* < 0.001). Likewise, with each point increase in the interpersonal communication metric, the adjusted odds of the abortion client reporting she was able to make her own choice of contraceptive method increases by 30% (aOR = 1.3, *p* < 0.01). Clients that experienced pressure to accept a particular contraceptive method have a 40% decreased odds of reporting the ability to make their own choice (aOR = 0.6, *p* < 0.05).

**Table 6:** Unadjusted and Adjusted Logistic Regression Models for Contraceptive Choice, Overall and by Age Category

Independent Variables	Unadjusted^a^			Adjusted^b^		
	**OR**	**95% CI**		**OR**	**95% CI**	
		**Lower**	**Upper**		**Lower**	**Upper**
	**All Clients (** *n* **= 1,969)**			**All Clients (** ***n*** **= 1,796)**		
Contraception Information Exchange Metric	2.1***	1.8	2.4	2.4***	2	2.9
Interpersonal Communication Metric	1.3**	1.1	1.5	1.3**	1.1	1.6
Client felt pressure from the provider to accept a particular contraceptive method	0.7*	0.4	1.0	0.6*	0.4	0.9
	**Clients Under 25 (** ***n*** **= 876)**			**Clients Under 25 (** ***n*** **= 779)**		
Contraception Information Exchange Metric	2.0***	1.6	2.5	2.5***	1.9	3.2
Interpersonal Communication Metric	1.2	1.0	1.5	1.2	0.9	1.6
Client felt pressure from the provider to accept a particular contraceptive method	0.7	0.4	1.2	0.6	0.3	1.2
	**Clients 25+ (** ***n*** **= 1,093)**			**Clients 25+ (** ***n*** **= 1,017)**		
Contraception Information Exchange Metric	2.2***	1.8	2.7	2.5***	2	3.2
Interpersonal Communication Metric	1.3*	1.0	1.7	1.5**	1.1	2.0
Client felt pressure from the provider to accept a particular contraceptive method	0.6	0.4	1.1	0.6	0.3	1.0

^a^Unadjusted models contain all three independent variables: Contraception information exchange metric, interpersonal communication metric and client felt pressure from provider to accept a particular method

^b^Adjusted models contain all three independent variables and client sociodemographic and abortion service characteristics are controlled. Client sociodemographic characteristics include country, marital status, age, education, and ever use of contraception. Abortion service characteristics included gestation, type of service and abortion procedure type.

* *p* < 0.05; ** *p* < 0.01; *** *p* < 0.001

Information exchange results are similar for models run by age group, but interpersonal communication only remains significant in the 25 + age group. Clients 25 + have a 50% increased odds of being able to make their own choice (aOR = 1.5, *p* < 0.01) for each 1-point increase in the interpersonal communication metric. In both models run by age group, pressure from providers to accept a particular contraceptive method is not significantly associated with the contraceptive choice outcome.

Table 7 provides the unadjusted and adjusted logistic regression models for whether the abortion client received a modern contraceptive method. This table only includes the main independent variables, but results for the sociodemographic variables were in expected directions (see Supplemental Table 3). With each additional element of information in the information exchange domain, the odds of the client receiving a method increases by 30% (aOR = 1.3, *p* < 0.001). This relationship is the same for clients under 25 (aOR = 1.3, *p* < 0.05), and clients 25+ (aOR = 1.3, *p* < 0.01). However, the interpersonal communication metric is not significantly associated with whether a client receives a method in either the full sample or age-specific samples. Abortion clients who felt pressure from the provider to accept a particular method had a 90% increase in odds in receiving a method (aOR = 1.9, *p* < 0.001). Disaggregating by age group reveals the same relationship for clients under 25 (aOR = 1.9, *p* < 0.05) and clients 25+ (aOR = 1.9, *p* < 0.01).

**Table 7:** Unadjusted and Adjusted Logistic Regression Models for Modern Contraceptive Method Received, Overall and by Age Category

Independent Variables	Unadjusted^a^			Adjusted^b^		
	**OR**	**95% CI**		**OR**	**95% CI**	
		**Lower**	**Upper**		**Lower**	**Upper**
	**All Clients (** ***n*** **= 1,972)**			**All Clients (** ***n*** **= 1,798)**		
Contraception Information Exchange Metric	1.2**	1.1	1.4	1.3***	1.2	1.5
Interpersonal Communication Metric	1.0	0.9	1.2	1.1	0.9	1.3
Client felt pressure from the provider to accept a particular contraceptive method	2.0***	1.4	2.8	1.9***	1.3	2.8
	**Clients Under 25 (** ***n*** **= 879)**			**Clients Under 25 (** ***n*** **= 781)**		
Contraception Information Exchange Metric	1.1	0.9	1.4	1.3*	1.1	1.7
Interpersonal Communication Metric	1.0	0.8	1.3	1.1	0.8	1.4
Client felt pressure from the provider to accept a particular contraceptive method	2.0*	1.2	3.3	1.9*	1.0	3.4
	**Clients 25+ (** ***n*** **= 1,093)**			**Clients 25+ (** ***n*** **= 1,017)**		
Contraception Information Exchange Metric	1.2**	1.1	1.4	1.3**	1.1	1.6
Interpersonal Communication Metric	1.0	0.8	1.2	1.1	0.9	1.4
Client felt pressure from the provider to accept a particular contraceptive method	2.0**	1.3	3.1	1.9**	1.2	3.0

^a^Unadjusted models contain all three independent variables: Contraception information exchange metric, interpersonal communication metric and client felt pressure from provider to accept a particular method

^b^Adjusted models contain all three independent variables and client sociodemographic and abortion service characteristics are controlled. Client sociodemographic characteristics include country, marital status, age, education, and ever use of contraception. Abortion service characteristics included gestation, type of service and abortion procedure type.

* *p* < 0.05; ** *p* < 0.01; *** *p* < 0.001

## Discussion

Assessing the quality of contraceptive counseling for abortion clients is important to ensuring abortion clients receive rights-based contraceptive care. We examined the quality of contraceptive counseling across two domains: information exchange and interpersonal communication, and tested two hypotheses: 1) that clients receiving higher quality contraceptive counseling will be more likely to receive a contraceptive method and feel empowered to make their own choice to accept or not accept a contraceptive method; and 2) that younger abortion clients receive lower quality contraceptive counseling and experience more pressure to accept a method than older clients. Overall, we found high quality contraceptive counseling in participating health facilities across both counseling domains. We confirmed that clients receiving higher quality contraceptive counseling, with respect to the information exchange domain, were more likely to receive a method. We also confirmed that clients receiving higher quality contraceptive counseling – across both domains – were more likely to make their own choice in accepting or not accepting a method. However, we found insufficient evidence to conclude that younger abortion clients received lower quality counseling or experienced more pressure to accept a method than older clients.

The contraceptive information exchange of postabortion contraception counseling was high with 84.2% (1,695 of 2,097) of abortion clients receiving four or more of the contraceptive information exchange elements included in the metric. Over 90% of abortion clients were asked about her desire to delay or prevent pregnancy, understood the information given to her about contraceptive methods, felt she was given enough information to choose a method, and was told that her fertility would return quickly. These findings suggest that providers at Ipas-supported facilities are providing women-centered contraceptive counseling that consider the needs and desires of abortion clients. These findings also demonstrate that providers are tailoring the contraceptive information in such a manner that women feel they have enough information and clear information. Additionally, the unique counseling information of abortion clients that their fertility will return quickly, was also routinely included during contraceptive counseling. However, inclusion of information on barrier methods to prevent STIs was the information most frequently omitted from counseling. This aspect of counseling is particularly important in countries with high rates of STIs, including HIV, and is an area for further study in future Ipas programming.

Ipas-supported facilities provided postabortion contraceptive care that met all five elements of the interpersonal communication metric for 78.1% (1,576 of 2,097) of abortion clients. As abortion clients often experience heightened stigma during care [23], this finding demonstrates that Ipas-supported facilities are treating abortion clients with dignity and respect, are giving clients the opportunity to ask questions and are listening to the needs and concerns that clients raise during counseling. Clients also felt they could trust the service providers to provide confidential care and felt they had sufficient privacy during their care.

The information exchange metric was significantly associated with having received a contraceptive method and having made her own choice to accept or not accept a method. Because abortion clients (including young abortion clients) experiencing an unintended and unwanted pregnancy often have little or poor knowledge about contraception [30], a strong emphasis on information exchange may be needed to improve informed contraceptive adoption for abortion clients. Additionally, our findings demonstrate the importance of adequate information exchange in supporting a woman’s right to choose to accept or not accept a contraceptive method which in turn can support abortion clients’ contraceptive autonomy.

The interpersonal communication metric was not significantly associated with having received a contraceptive method, and this was consistent in the models for youth and non-youth. However, our findings do show that interpersonal communication during postabortion contraceptive counseling is important in fostering a perception of contraceptive method choice among abortion clients. Abortion clients scoring higher in the interpersonal communication metric had a significantly higher odds of feeling they were able to make a choice of contraceptive method (or no method). There is strong evidence that interpersonal communication is important for client satisfaction with their contraceptive counseling and service [30]. This echoes Pilgrim et al.’s 2014 paper, that also found an association between autonomous contraceptive decision-making and the quality of contraceptive care: clients that reported making their own contraceptive decision were more likely to report receiving high-quality contraceptive services [31].

Although only 14% of abortion clients counseled on contraception reported being pressured from a provider to accept a particular contraceptive method, our results show that pressure from the provider doubled the odds of an abortion client receiving a contraceptive method. Inversely, the existence of pressure from the provider reduced the odds by 40% of a client feeling she had made her own choice of contraceptive method. While these results may imply coercive behaviors among some providers towards a minority of abortion clients, the lack of direct observation in this study limits our ability to concretely conclude the presence of subtle or overt coercion. Further exploration is needed to better understand how coercive behaviors from providers, like pressure to accept a contraceptive method, manifests for abortion clients in LMICs as studies with abortion clients in the United States have found evidence of provider pressure [32, 33]. This finding also demonstrates the impact pressure from a provider can have on clients, increasing method acceptance but decreasing autonomy and choice. Numerous studies with family planning clients, including postabortion clients [34], have demonstrated that clients prefer to make the contraceptive decision themselves, and highly value autonomy in contraceptive decision-making [35, 36]. Having control over the ultimate selection of a contraceptive method is not only important to client satisfaction but is likely a contributing factor to continued use of the selected method [16, 37]. Cardona et al. found that contraceptive service client’s satisfaction with their service was significantly associated with continuation of the method among family planning clients in three sub-Saharan African countries [37]. Postabortion contraceptive counseling that seeks to provide high-quality information exchange and interpersonal communication can empower women to choose the method that is best for their needs and preferences.

We could not conclude any significant differences in the counseling quality metrics and the associations of quality counseling to method receipt or choice for youth compared to non-youth. Youth had a marginally higher mean score on the information exchange metric largely due to young abortion clients more often being told about a barrier method for protection against STIs compared to non-youth clients. Although our study did not find any discernable difference between youth and non-youth, there is strong evidence in LMIC that demonstrates that young people, in particular adolescents, have limited knowledge of contraceptive methods [38]. Youth often have misconceptions about the side effects of contraceptives on their health and future fertility [39–41] and poor understanding of how contraceptives work and how they should be used [39, 42]. The experiences of adolescent abortion clients may be unique but, unfortunately, our sample size of adolescent abortion clients was not sufficient to compare quality of care among adolescents and non-adolescents.

These findings also reveal significant differences in the receipt of postabortion contraceptive counseling across various abortion service characteristics. Although a high proportion of abortion clients in our sample (84.3%, 2,097 of 2,488) did receive contraceptive counseling during their visit in the facility for abortion services, PAC clients and clients with gestations at or after 13 weeks were less likely to receive postabortion contraceptive counseling than induced abortion clients and clients with gestations under 13 weeks (79.1% of PAC clients versus 91.0% of induced clients received contraceptive counseling; 74.6% of clients with gestations at or after 13 weeks versus 85.6% of clients with gestations under 13 weeks received contraceptive counseling). The lower rate of contraceptive counseling among PAC clients is consistent with other studies [43, 44] and may reflect multiple factors. First, some women seeking PAC and with gestations at or after 13 weeks may have desired pregnancies and wanted to become pregnant again soon after their service, and therefore may have not desired any contraceptive counseling. Second, a disproportionate share of PAC is provided in secondary and tertiary facilities, where PAC may not be well-linked with contraceptive services [43]. Thirdly, some PAC clients might have received emergency care in our sample, and this could contribute to the lower contraceptive counseling rates as contraceptive counseling after an emergency service might not be done routinely. Additionally, provider biases and disinclination to offer contraception after PAC [45] and with clients with gestations at or after 13 weeks may be present. In some settings, especially countries with the most legally restrictive abortion laws, abortion services are predominantly PAC. These clients, as well as clients with gestations at or after 13 weeks, can face heightened abortion stigma that may affect the willingness of the health worker to provide contraceptive counseling to these clients. These finding echoes previous studies that have found contraceptive service provision to PAC clients [46, 47] and clients with gestations at or after 13 weeks [44] to be low.

### Limitations

The limitations of this study include potential courtesy bias, as abortion clients may be more hesitant to share any negative experiences involving the care they received while still in the facility in which they received care. We tried to mitigate this by conducting interviews with trained interviewers who were not involved in the client’s care nor employed by the facility. Additionally, when possible, interviews were conducted in spaces that provided visual and auditory privacy.

Another limitation is the challenge of capturing complete contraceptive service and counseling data from abortion clients that received medical abortion. In many settings, MA clients receive their first dose of medical abortion in the health facility but finish the abortion at home. Because many of these clients may not have completed their abortion before leaving the facility and at the time of their interview, it might not be an appropriate or ideal time to provide contraceptive counseling with clients. Some contraceptive methods cannot be provided to the client until she has completed her abortion, such as IUCD and sterilization. In these cases, women must return to the health facility after the abortion is complete to receive these types of contraceptive methods.

Another limitation of this work is the potential selection bias of excluding abortion clients that indicated they did not receive any information on contraceptive methods. In this analysis, we excluded these clients from the analysis of the quality of contraceptive counseling; this represented 16% of abortion clients interviewed. A portion of these clients who did not receive postabortion contraceptive counseling might have been interviewed before they received their contraceptive care, as indicated previously. However, about a quarter of these clients who did not receive contraceptive counseling did report having received a contraceptive method at the facility. This finding could indicate that some clients requested a contraceptive method and did not desire any counseling about the method from the provider, or that health workers provided a contraceptive method without performing any counseling about the method. Without further study, we are unable to know the reasons some clients received contraceptive methods without any counseling.

In our survey instrument, we asked about the client’s fertility intentions but did not ask clients if they desired a contraceptive method during the interview. In future client exit interviews with abortion clients, we hope to better capture client’s desire for any contraceptive method. Finally, we were unable to recover unique facility identifiers in the data from Nepal and Ethiopia. Due to this, we were unable to perform an analysis that adjusts for site clustering using the full sample. However, sensitivity testing using data from the six countries where facility identifiers were available suggested that clustering did not significantly influence the results.

## Conclusion

Ipas-supported facilities provided high rates of high-quality contraceptive counseling and service provision after abortion services across all study countries. We found that the quality of contraceptive counseling, including the information exchange and interpersonal communication between the client and provider, to be crucially important for ensuring that clients feel they made their own choice of contraceptive method. Although a small proportion of clients experienced pressure and lack of choice in Ipas-supported health facilities, we hope to further examine these aspects of care to develop strategies to ensure contraceptive autonomy for abortion clients. We also hope to strengthen our measurement of clients’ perspective of the care experience, including developing indicators to better monitor contraceptive autonomy for abortion clients. During postabortion contraceptive counseling in health facilities, it is vitally important that programs continue to foster informed choice through strengthening interpersonal communication and contraceptive information exchange during counseling. This will ensure postabortion contraceptive services are woman-centered and rights-based for all abortion clients.

## Supplementary Information

**Additional file 1: Supplemental Table 1.** Sensitivity Testing of Adjusted Logistic Regression Models for Contraceptive Choice and Receipt of a Contraceptive Method. **Supplemental Table 2.** Adjusted Logistic Regression Models for Contraceptive Choice, Overall and by Age Category. **Supplemental Table 3.** Adjusted Logistic Regression Models for Modern Contraceptive Method Received, Overall and by Age Category.

## Data Availability

The datasets used and analyzed for this paper are available from the corresponding author, Valerie N. Acre (acrev@ipas.org), on reasonable request. The informed consent process for this study did not include informing potential participants that de-identified datasets would be made publicly available online. We will happily make the dataset available upon request, but we wish to adhere to the original informed consent process approved by each IRB.

## Declarations

The data collection of the monitoring and evaluation data presented in this paper was submitted and approved by local ethics committees in Argentina, Ethiopia, Nepal, and Nigeria; and was submitted and approved by a US-based ethics committee for Argentina, Bolivia, and Mexico. For Bolivia and Mexico, a US-based ethics committee was used due to the researchers’ inability to identify an appropriate local ethic review board to review this study. Ethical approval was obtained from 1) the Allendale Institutional Review Board in the US for client exit interviews in Argentina, Mexico and Bolivia (IRB No.: Ipas_LAC_CEI_10Dec2018); 2) the Ethics Committee of the Ministry of Health of the City of Buenos Aires (IRB ID: “Entrevistas de Salida con usuarias de servicios de aborto legal y posaborto en instituciones de la red publica de salud en Argentina, Version 1, 25-abril 2019”) and facility level committees of bioethics from participating health facilities in Argentina; 3) the Amhara Public Health Institution (IRB No.: H/RTTD/1/473) and Oromia Health Bureau (IRB No.: BEFO/HBTFU/116/120) in Ethiopia; 4) the National Health Research Ethics Committee in Nigeria (IRB No.: NHREC/01/01/2007-29/04/2019); and 5) Nepal Health Research Council in Nepal (IRB No.: 1726, Reg. No. 8/2018). In Kenya and Uganda, Ipas and sub-regional ministries of health partners in each country did not seek review from a local ethical review committee because the purpose of the data collected was for routine monitoring and evaluation data of Ipas and MOH programming, and not for research.

Written informed consent was obtained for all participants in Ethiopia, Nepal, Kenya, Nigeria, and Uganda. Verbal informed consent was obtained in Argentina, Bolivia, and Mexico. Verbal consent was documented by the interviewer through the mobile application for comfort and ease of respondents rather than using electronic signatures on mobile devices. Verbal consent procedures were approved by the Allendale Institutional Review Board in the US, the local Ethics Committee of the Ministry of Health of the City of Buenos Aires and facility level committees of bioethics from participating health facilities in Argentina.

According to Law 27337 Article 21 of Bolivia’s “Code for Children and Adolescent” and the “Technical and Procedural Guidelines for Safe Abortion Services in Ethiopia, Second Edition” from the Federal Democratic Republic of Ethiopia Ministry of Health, women under the age of 18 in Ethiopia and Bolivia may request and receive abortion services without the consent of a parent or guardian. We received a waiver from the Allendale Institutional Review Board in the US for the interviews in Bolivia, and from the Amhara Public Health Institution and Oromia Health Bureau for the interviews in Ethiopia to allow minors in these countries to decide to participate in an SRH-related study without parental or guardian consent.
